# Delayed gangrenous penile necrosis following prolonged utilization of penile constriction ring

**DOI:** 10.1016/j.eucr.2024.102762

**Published:** 2024-06-01

**Authors:** Vi Nguyen, Nishant Garg, Benjamin E. Cedars, Dhruv Puri, Kian Ahmadieh, Jill C. Buckley

**Affiliations:** Department of Urology, University of California, San Diego, La Jolla, CA, USA

**Keywords:** Penile necrosis, Penile constriction ring, Split thickness skin graft, Penile reconstruction, Gangrene

## Abstract

Penile strangulation secondary to utilization of a constrictive ring is a rare urologic emergency that requires urgent decompression to prevent prolonged vascular obstruction resulting in necrosis and gangrene. Current literature is mainly comprised of case presentations that focus on management in the acute setting via removal of the ring. Herein, we describe surgical management of a patient who presents in delayed fashion after self-removal of the constrictive ring. We discuss our penectomy-sparing technique of debridement and split thickness skin graft.

## 1 Introduction

Penile necrosis and incarceration secondary to utilization of a constrictive ring is a rare urologic emergency.^1^ First described in 1755, penile constrictive rings are intended to prolong erection for sexual enhancement by reducing outflow of blood.^2^ In the pediatric population they may be rarely used for enuresis or misapplied newborn circumcision devices, and therefore prone to being left on for an extended period of time.^3^ Patients with penile entrapment often present with penile edema and pain.^1^^,^^2^^,^^4^, ^5^, ^6^ Risk factors for entrapment include utilization of a metal ring, improper ring sizing, and prolonged use which can result in penile ischemia, necrosis, and urinary retention.^4^ Common reasons for delayed presentation include patient embarrassment, a poorly controlled psychiatric history, or possible drug and alcohol use.^7^

Given the rare presentation, current literature is largely comprised of case presentations or series. These studies are often focused on different techniques aimed at ring removal in the emergency setting as well as maintenance of urinary function, with little attention paid to cosmesis and delayed functional recovery.^8^ Various devices that have been described include chisels, orthopedic saws, grinders, and dental drills.^2^ Copious irrigation and application retractors are critical to preventing thermal injury.^4^

Management of patients who present in a delayed fashion has been described less frequently due to the acute nature of this clinical issue. Herein, we describe surgical management of a patient who presents in a delayed manner with sequelae of prolonged penile constriction after self-removal of the ring.

## 2 Case presentation

This is a 68-year-old male who presented with penile pain and swelling after prolonged utilization of a penile constrictive ring. Per patient report, the ring was in place for 24 hours prior to self-removal with pliers. The patient presented to an outside Emergency Department (ED) due to difficulty urinating.

**Fig. 1:**
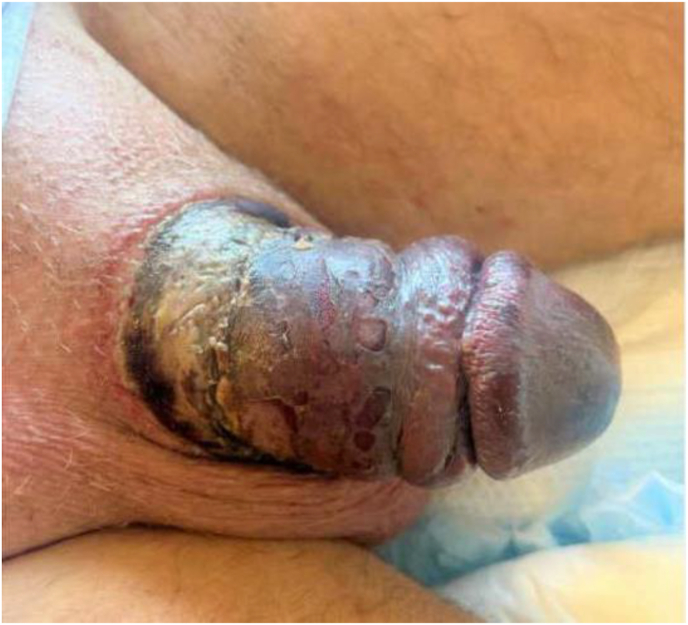
Patient's penile examination on initial presentation.

He was then transferred to our hospital 4 days later for urologic care. On arrival, he was hemodynamically stable and laboratory workup did not reveal any abnormalities. Genitourinary examination revealed erythematous and blistered skin (Fig. 1). A foley catheter was placed by Urology without difficulty. Given patient's clinical stability and his presentation one week after initial injury, no acute intervention was deemed necessary. He was discharged to a nursing facility with the assistance of Social Work given his housing insecure status with plan for close outpatient follow up.

**Fig. 2:**
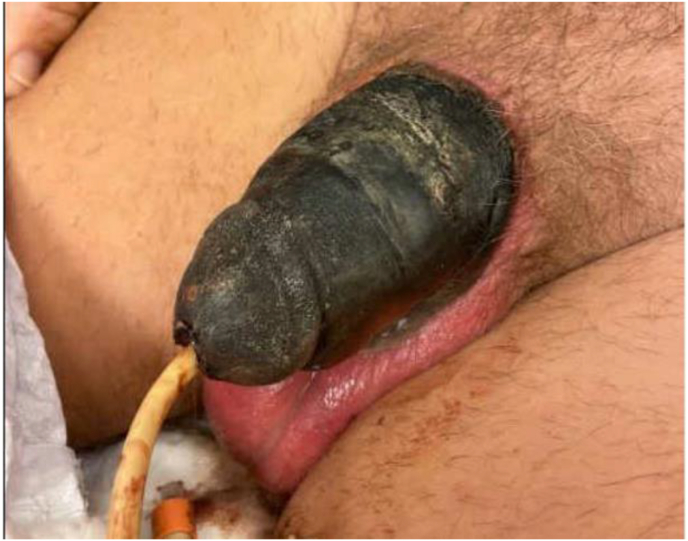
Worsened examination consistent with penile necrosis after presentation to clinic.

He then presented to clinic 2 weeks later with significant interval change in examination demonstrating black necrotic tissue concerning for gangrene (Fig. 2). His catheter from prior admission was still in place and draining without issue. He was sent to the ED from clinic and admitted to the Urology service. The patient was immediately started on intravenous broad-spectrum antibiotics (vancomycin, piperacillin/tazobactam).

**Fig. 3:**
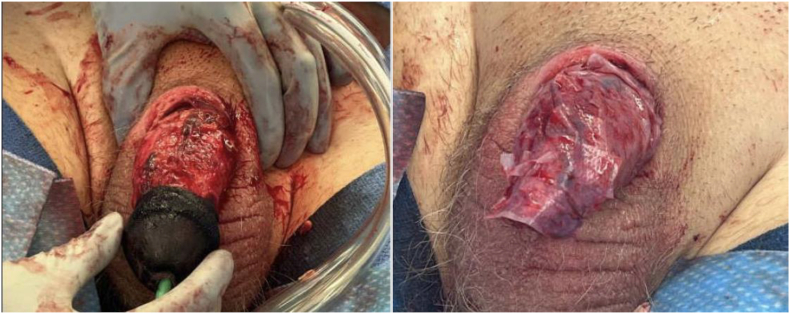
Intraoperative findings on initial debridement.

The following day, he was taken to the operating room. Cystoscopy demonstrated necrosed distal urethra; the urethra appeared healthier starting at the bulb and proximally beyond. A 16Fr suprapubic tube was placed under direct cystoscopic visualization. Exam under anesthesia revealed dry gangrene with necrotic eschar on the dorsal aspect of the phallus and wet gangrene on the ventral aspect. The entire shaft was debrided circumferentially sharply using a combination of the 15 blade and Metzenbaum scissors until viable tissue was encountered (Fig. 3). Several purulent corporal pockets on the ventral aspect were bluntly explored and irrigated. Corporal hemostasis was achieved with simple interrupted 3-0 PDS. A small area of erosion at the distal urethra was repaired with simple interrupted 5-0 PDS. The entire glans was replaced with thick woody eschar; thus, the decision was made to completely excise this portion. Urinary bladder matrix (ACell, Integra LifeSciences Corporation, San Diego, CA, USA) was then applied circumferentially on the shaft and secured with simple interrupted 4-0 chromic suture (Fig. 3). A dressing of xeroform, kling, and coban was applied to conclude the case.

The patient remained admitted to the Urology service for observation and antibiotics given his tenuous housing situation. He remained clinically stable during this time and was taken back to the operating room on postoperative day (POD) 7 for repeat debridement and skin grafting.

**Fig. 4:**
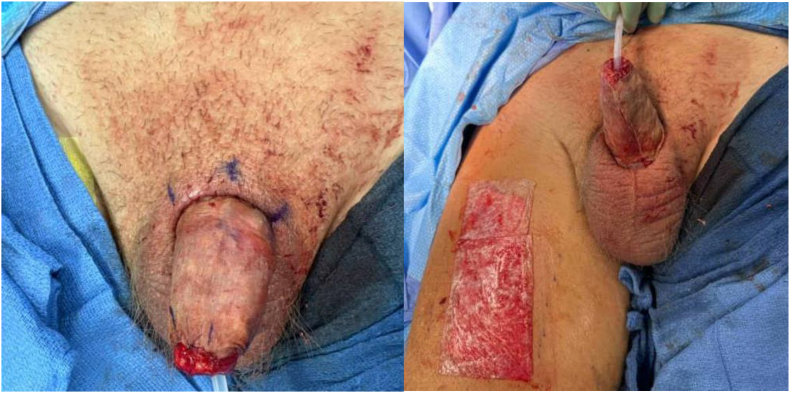
Application of split thickness skin graft.

Intraoperative examination revealed a small 2cm area of necrosis on the left corpora that was sharply excised. The remainder of the penile shaft appeared much healthier compared to the prior surgery and minimal debridement was performed until healthy viable tissue was encountered. The prior area of distal urethral erosion was the sharply incised to create a megameatus to avoid future possible fistulization. The penile shaft was then measured, and attention was turned to the right medial thigh. An 8 × 13cm split thickness skin graft (STSG) was harvested using the dermatome (1/18,000 inch). The graft was then laid circumferentially around the shaft and tacked into place using simple interrupted 3-0 chromic sutures (Fig. 4). The donor site was dressed with ACell wound matrix and xeroform (Fig. 4). A bolster dressing was then applied. First, we placed circumferential 4-0 Monocryl sutures at the base proximally and the tip of the shaft distally. Next, the graft was wrapped with xeroform and mineral oil-soaked cotton balls. This was then wrapped with kling. 4 × 4 fluffed gauze was placed circumferentially. A clean green light handle with the covering removed was then placed over the dressing and secured with a 3-0 nylon suture in the scrotal skin superficially over a tongue blade.

The patient remained clinically stable after surgery and remained inpatient until POD5/12 when his dressing was taken down. With the assistance of case management, he was safely discharged to a skilled nursing facility.

He was evaluated in clinic POD 12/19. The STSG was noted to be healing well with approximately 85 % take. The suprapubic catheter has been draining without issue. He will continue to be followed up closely with routine clinic visits.

## 3 Discussion

Penile strangulation from a constrictive ring is a urologic emergency that warrants immediate removal to prevent vascular compromise and subsequent necrosis, as well as acute urinary retention. Management of these patients poses a clinical challenge given availability of tools available to the urologist at their respective facility to remove the constrictive device.^9^^,^^10^ Prompt treatment is critical to preventing sequelae of prolonged vascular congestion including necrosis, gangrene, and autoamputation. Left untreated, delayed complications include decreased/loss of penile sensation, urethral stricture, and urethral fistula.^11^

Severity of penile strangulation has been stratified by Bhat et al., in 1991, and is summarized in Table 1^12–14^. According to this classification, our patient suffered a Grade V injury given development of necrosis and gangrene. Previously, these patients were most likely managed with partial penectomy.^12^, ^13^, ^14^ Herein, we describe management of our patient with debridement and STSG in efforts to spare penile amputation.

**Table 1:** Grading scale for penile incarceration.

Grade	Clinical Description
*I*	Edema of distal penis
	No evidence of skin ulceration or urethral injury
*II*	Injury to penile skin
	Constriction of corpus spongiosum without urethral injury
	Decreased distal penile sensation
*III*	Skin or urethral injury without urethral fistula
	Loss of distal penile sensation
*IV*	Complete division of corpus spongiosum leading to urethral fistula
	Constriction of corpus cavernosa with loss of distal penile sensation
*V*	Gangrene, necrosis, or complete amputation of distal penis

The utility of STSG for male genitourinary reconstruction has been described for a variety of underlying conditions including tissue loss (i.e. post-Fournier's gangrene), lymphedema, buried penis repair, foreign body injection, hidradenitis suppurativa, malignancy, condyloma acuminatum, and extramammary Paget's disease.^15^, ^16^, ^17^ To our knowledge, this is the first description of successful utilization of this technique for tissue loss following debridement of penile necrosis secondary to use of a constrictive ring. Further follow up is needed to evaluate this patient's long-term outcomes.

## 4 Conclusion

Penile necrosis and gangrene secondary to prolonged utilization of a constrictive ring is a rare urologic emergency. Though these patients often present in the acute emergency setting, we describe successful management of a patient who presents in a delayed manner with Grade V penile incarceration using an amputation-sparing surgical technique of debridement and STSG.

## CRediT authorship contribution statement

**Vi Nguyen:** Conceptualization, Data curation, Writing – original draft, Writing – review & editing. **Nishant Garg:** Writing – original draft, Writing – review & editing. **Benjamin E. Cedars:** Writing – review & editing. **Dhruv Puri:** Writing – review & editing. **Kian Ahmadieh:** Writing – review & editing. **Jill C. Buckley:** Conceptualization, Supervision, Writing – review & editing.
